# Design and Implementation of a Smart Home in a Box to Monitor the Wellbeing of Residents With Dementia in Care Homes

**DOI:** 10.3389/fdgth.2021.798889

**Published:** 2021-12-21

**Authors:** Matias Garcia-Constantino, Claire Orr, Jonathan Synnott, Colin Shewell, Andrew Ennis, Ian Cleland, Chris Nugent, Joseph Rafferty, Gareth Morrison, Leona Larkham, Sharon McIlroy, Andrea Selby

**Affiliations:** ^1^School of Computing, Ulster University, Jordanstown, United Kingdom; ^2^The Lava Group, Belfast, United Kingdom; ^3^Kirk House Care Home, Belfast, United Kingdom

**Keywords:** connected health, ambient assisted living, smart homes, ubiquitous computing, pervasive computing, dementia, care home

## Abstract

There is a global challenge related to the increasing number of People with Dementia (PwD) and the diminishing capacity of governments, health systems, and caregivers to provide the best care for them. Cost-effective technology solutions that enable and ensure a good quality of life for PwD *via* monitoring and interventions have been investigated comprehensively in the literature. The objective of this study was to investigate the challenges with the design and deployment of a Smart Home In a Box (SHIB) approach to monitoring PwD wellbeing within a care home. This could then support future SHIB implementations to have an adequate and prompt deployment allowing research to focus on the data collection and analysis aspects. An important consideration was that most care homes do not have the appropriate infrastructure for installing and using ambient sensors. The SHIB was evaluated *via* installation in the rooms of PwD with varying degrees of dementia at Kirk House Care Home in Belfast. Sensors from the SHIB were installed to test their capabilities for detecting Activities of Daily Living (ADLs). The sensors used were: (i) thermal sensors, (ii) contact sensors, (iii) Passive Infrared (PIR) sensors, and (iv) audio level sensors. Data from the sensors were collected, stored, and handled using a ‘SensorCentral’ data platform. The results of this study highlight challenges and opportunities that should be considered when designing and implementing a SHIB approach in a dementia care home. Lessons learned from this investigation are presented in addition to recommendations that could support monitoring the wellbeing of PwD. The main findings of this study are: (i) most care home buildings were not originally designed to appropriately install ambient sensors, and (ii) installation of SHIB sensors should be adapted depending on the specific case of the care home where they will be installed. It was acknowledged that in addition to care homes, the homes of PwD were also not designed for an appropriate integration with ambient sensors. This study provided the community with useful lessons, that will continue to be applied to improve future implementations of the SHIB approach.

## Introduction

There is a global challenge with an increase in the number of People with Dementia (PwD). The World Health Organization (WHO) has projected a global increase in PwD from 50 million in 2021 to 152 million by 2050 (1). A report commissioned by the Alzheimer's Society from the Care Policy and Evaluation Center (CPEC) at the London School of Economics and Political Science (LSE), showed that as of 2019 there were an estimated 885,000 people living with dementia in the UK (2). While advances in medicine have contributed to a global increase of life expectancy, chronic conditions in older adults (including those with dementia) have represented a challenge to the capacity of governments, health systems, and caregivers to provide the best care for them (3). Cost-effective technology solutions that enable and ensure a good quality of life for monitoring older adults for prompt interventions have been investigated comprehensively in the literature.

PwD are typically aged 65 years or older (4), which adds other risk factors that health professionals need to consider, such as: (i) lack of self-sufficiency in everyday life (for example, needing help from carers for dressing or eating) (5), (ii) having other chronic diseases (6), (iii) the burden on family caregivers (7), (iv) the caregiver's technological literacy, and (v) the PwD's acceptance of technology (8). The rapid development of sensor technology and its affordability have enabled researchers in the areas of Computer Science, Healthcare, Ambient Assisted Living (AAL) and Pervasive Healthcare to find cost-effective solutions that can provide support for caregivers of PwD, which in relation to other populations in general (not just with dementia), are overworked and understaffed (9).

Activity recognition is the detection, interpretation, and recognition of human activities using sensors, either ambient or embedded, in the environment or worn on the body. In addition to supporting the wellbeing of PwD by monitoring their activities to detect abnormal behaviors that could negatively impact their health, activity recognition also supports the work of caregivers by relieving some of their work load so they can provide better care to PwD. Note that caregivers can be formal (working in care homes or health institutions, typically understaffed) or informal (usually relatives of PwD who live with them).

Embedded sensors include those that are incorporated into a device such as a mobile phone, watch, or into clothing (10). While these wearable sensors have the advantages of monitoring and collecting data regardless of the users' location, they have some disadvantages related to privacy, security, robustness, accuracy, and technology-dependency of the users (11). Ambient sensors are attached to objects in the environment with which the user interacts (e.g., door, kettle, ceiling, walls). These types of sensors have the advantages of not being intrusive and typically do not require regular maintenance, such as charging (12). However, the main disadvantages of environmental sensors can be inability to function adequately according to how well placed they are in the environment, and they also present challenges in monitoring multiple occupants in the environment. Such issues could include hardware issues (e.g., malfunction, unreliable connection to the Internet), physical damage, or loss of power/energy (13). Another aspect to consider is how accurate can wearable and embedded sensors can be (14, 15).

The main motivations of this study are in the context of supporting PwD and their carers to monitor and identify unusual activities that may affect the well-being of PwD. Previous work in the literature has been investigating the use of sensors for quantification of well-being (16, 17). The objective of this study is to investigate the challenges and opportunities in the stages of design, deployment, and functioning of a Smart Home In a Box (SHIB) approach in a dementia care home environment; to monitor the wellbeing of PwD as well as to identify lessons that could support improved future implementations of the approach. Note that the focus of this study is on dense sensors placed in a care home, therefore the use of wearable sensors was not investigated in the case study. It was expected to install sensors in the rooms of service users within dementia units, delirium units, or intermediate care.

The remainder of the paper is organized as follows: Section Literature Review presents the related work in the area of using Smart Home approaches for activity recognition. Section Methods describes the design of the SHIB approach proposed and its implementation in a care home. Section Results presents the challenges, opportunities and lessons learned from the installation and utilization of the SHIB in a care home. Section Discussion presents a discussion on the findings of the study, and Section Conclusions and Future Work presents conclusions and future work.

## Literature Review

While the focus of this study is on the technical and usability aspects of implementing a SHIB approach, it is worth noting some of the ethical aspects related to Smart Home design that were investigated in the literature. Chung et al. (18) state that the ethical considerations in Smart Home design are:

(i) Privacy: refers to informational privacy and the right of the users to control the access and sharing of their personal data.
(ii) Informed consent: refers to the description of the objectives of the Smart Home research, details about its implementation, and the potential benefits and risks related to the use of the technologies involved.
(iii) Autonomy: involves the users' sense of independence supported by the automation of the technologies used.
(iv) Lack of human touch: refers to the decrease in human interaction derived from the use of virtual visits or remote monitoring.
(v) Medicalization of the home environment: relates to a major focus of health within the Smart Home environment.
(vi) Obtrusiveness: refers to the users' perception of aspects of the technologies used as physically and psychologically noticeable.
(vii) Equity of access: means having universal access to necessary health information and means for monitoring it.
(viii) Usability: refers to the way users use the technology to perform tasks in the Smart Home environment.

In the work presented by Birchley et al. (19), 20 smart home researchers were interviewed about ethical considerations related to smart home design and it was found that the two major themes were: (i) privacy, and (ii) end-user choices. In this case, it was mentioned in (19) that concerns about privacy might reduce the acceptability of Smart Home technologies. End-user choices refers to how providing users with choices on when and how to use Smart Home technologies to address ethical considerations may affect the effectiveness of the Smart Home. More recently than these, Hall & Maglaras (20) wrote about the security challenges and privacy concerns of smart homes. Here they also highlight that the main privacy issues are: (i) cloud storage by third parties, (ii) secondary use of data collected from devices such as Smart Speakers, and (iii) attacks/hackers.

### A Review of Smart Home Approaches in Dementia Care Homes

This subsection presents related work about smart home approaches in dementia care homes categorized as: (i) traditional smart home approaches, and (ii) SHIB approaches. The main distinction between these approaches is that, in the former, many modifications would be required to the care home. Whereas, in the case of the latter one, minimal or no modifications are needed. Note that in some cases the approaches presented do not refer specifically to the case of smart environments monitoring dementia, but to their capabilities, including monitoring activities, which could be adapted for a health context. Table 1 presents a summary of the Smart Home approaches (traditional and SHIB) investigated from the literature and the sensors that were respectively used. The variety of scenarios and types of sensors included in the Smart Home approaches revised in this section show how there is no generic or standardized approach and that in most cases they seemed to be tailored to address specific needs from the elderly users.

**Table 1:** Smart Home approaches and sensors included.

**Name of project**	**Application type**	**Sensor type**	**Parameter of interest**
Georgia-Tech Aware Home (21)	Traditional	RGB camera Piezoelectric force-sensitive floor tiles	Facial recognition Gait/Footstep patterns and location
MavHome (Managing an Adaptive Versatile Home) (22)	Traditional	Robotics Mobile computing	Mobility patterns *via* machine learning Device usage (23)
Gator Tech Smart Home (24)	Traditional	Ultrasonic sensors Contact sensors Temperature sensors	Location Movement Orientation Temperature
ACHE (adaptive control of home environment) Smart House (25)	Traditional	Temperature sensors Actuator nodes	Temperature Lighting
“Smart house in a box” at Gator Tech Smart House (26)	SHIB	Pressure sensors Moisture sensors Actuator nodes Ultrasonic transceivers	Pressure Moisture Light
CASAS (27)	SHIB	Contact sensors	Temperature Motion
HINT (Halmstad Intelligent Home) (28)	SHIB	Contact PIR Pressure	Physiological, safety, functional and emergency monitoring
Dem@Home (29)	SHIB	Ambient cameras Wireless tags Presence sensors	Image and depth Activity levels Sleep
EurValve Smart Home in a Box (30)	SHIB	Video Wearable (Accelerometer)	Image Movement

### Traditional Smart Home Approaches

Traditional Smart Home approaches relate to the cases where many modifications must be done in the care home, which could result in additional costs due to the sensors required to be installed. While there has been an increase in the acceptance and use of health tracking devices (blood pressure monitors, activity trackers, blood glucose trackers, *etc*.) by users at their homes or at care homes, dedicated modifications to buildings have only been done at hospitals or at specialized private care homes. The main reasons for this are the costs involved in modifying an individual's home and the required basic technical knowledge to operate health devices or specialized equipment installed at their homes. A traditional Smart Home approach for a care home will involve: (i) planning from the design stage of the building, or (ii) carrying out extensive modifications to an existing building.

The “Aware Home” prototype presented in (31) was intended as a living laboratory for research in ubiquitous computing for everyday activities and included the following spaces in the house: two identical and independent living spaces, consisting of two bedrooms, two bathrooms, one office, kitchen, dining room, living room, and laundry room. One of the living spaces was intended to be where the person would spend most of the time during the duration of the study. Although the authors in the original publication (32) mentioned that the “Aware Home” could be used for general purposes. The specific application of “support for the elderly” is presented with three main areas of interest: (i) social connections between elderly parents and their adult children, (ii) support for “everyday cognition” by supporting memory decline and planning capabilities, and (iii) identification of potential crisis situations and contact of appropriate services to act upon them.

A Smart Home approach called “MavHome” (33) (Managing an Intelligent Versatile Home) operates as an intelligent agent that identifies the states of the home using sensors and acts on the situation using device controllers (34). The “MavHome” agent includes technologies distributed in four layers that cooperate with each other: (i) decision (selects actions to be executed), (ii) information (gathers, stores, and generates knowledge for decision making), (iii) communication (communicates information, requests, and queries between agents), and (iv) physical (contains the hardware equipment including individual devices, transducers, and network hardware) (35). Sensors within this home monitor the environment and then use the communication layer to transmit the information to another agent (36). While the use of the “MavHome” for monitoring health of elderly people or PwD is not indicated explicitly, the capabilities described indicate that it could be used for those purposes.

The “Gator Tech Smart Home” approach had the goal of creating assistive environments that could sense and monitor themselves and their occupants allowing for intervention services (37). This project has a generic architecture that could be applicable to pervasive computing spaces in general and includes the following layers: (i) physical, (ii) sensor platform, (iii) service, (iv) knowledge, (v) context management, and (vi) application (38). In a publication by L. Y. Mano (39) the “Gator Tech Smart Home” is aimed at monitoring the health of and assisting elderly people, by creating supportive environments and developmental activities. The goal of this was to maximize independence of the residents and maintain a high quality of life. The authors of (37) mention that their goal is to create a SHIB approach that can be bought, installed, and monitored by the end users without the support of engineers.

The objective of the ACHE smart house was to monitor the environment, observe then predict the actions of the user. From this the energy consumption of the house could be controlled, for example, lighting, air temperature, ventilation, and water heating (40, 41). Equipped with more than 75 sensors and actuators, this environment had the benefit of reducing the overall energy consumption whilst maintaining the user's comfort. To achieve this objective, the ACHE system used Reinforcement Learning (RL), where a high-level environmental state is built from the sensor data recorded and a controller then makes decisions based on the current activity and location of the user (42).

### Review of Smart Home in a Box Approaches

SHIB approaches relate to the cases where minimal or no modifications must be done to an existing care home. End users should also be able to install and configure the equipment and sensors included in the SHIB by themselves, with minimal or no support from technical staff (43). The mechanisms to install and uninstall the sensors included in SHIB in a building should be straightforward for non-technical users. One of the expected main advantages of SHIB approaches based on our research is that costs are reduced by: (i) not making structural modifications to the building, (ii) not requiring extensive training or support by technical staff to install/uninstall and use the sensors included in a SHIB, and (iii) by re-using the equipment when necessary (i.e., if one room becomes unoccupied and another resident deteriorates, the sensors can be moved as needed rather than buying more). A SHIB approach for a care home will involve: (i) completing a building survey to know the most adequate places to install/uninstall sensors, and (ii) include clear and intuitive instructions on how to install/uninstall and use the sensors in a SHIB.

Abdulrazak and Helal (44) presented a dedicated SHIB approach that focuses on an easy integration and management of assistive space to support aging people with daily activities. It is mentioned that the SHIB presented: (i) needs minimum engineering expertise, (ii) facilitates the system integration phase, and (iii) reduces the cost of deployment. It is also mentioned that it can be integrated with the “Gator Tech Smart House (26). In addition to the SHIB approach, the authors of (44) also present the “Gator Tech Smart House” (GTSH) architecture, which is described as a generic architecture that can be implemented in any pervasive computing space. They note that it is challenging and time consuming to have a proper integration of the sensors that are part of a SHIB approach, thus their solution is in the form of plug-and-play mechanisms for a better sensor integration. Their self-integration framework is comprised by two parts: (i) hardware framework [Atlas Platform (45)], and (ii) software framework [middleware built on top of the Open-Source Gateway Initiative (OSGi)]. In this case the SHIB approach can be integrated with the “Gator Tech Smart House,” and therefore it is possible to use the related sensors. The use of the GTSH architecture allows integration with other types of sensors. The capabilities described for this SHIB approach would allow its use to monitor the health of elderly people or PwD.

A well-established SHIB approach is the CASAS (Center for Advanced Studies on Adaptive Systems) architecture that was presented by Cook et al. (46), which presents to the users a box which includes the sensors and equipment to be installed. The objective of the CASAS SHIB approach is to present a small, lightweight, extendable, and user-friendly SHIB that is ready to function out of the box. It is stated that the capabilities of the CASAS SHIB system are: (i) activity recognition (mapping a sequence of sensor data to an activity label), (ii) activity discovery (identify activities from streaming data), and (iii) activity-aware applications (presenting prompts to the users that are related to specific activities based on personalized patterns). The CASAS software included in the SHIB comprises: (i) a machine learning algorithm to identify personalized patterns on users' daily activities, and (ii) an activity visualizer that can be shown on a computer or on a mobile device. The activities considered for the activity recognition functionality of the CASAS SHIB are: (i) bed-toilet transition, (ii) cooking, (iii) eating, (iv) entering home, (v) leaving home, (vi) personal hygiene activities, (vii) using the phone, (viii) relaxing, (ix) sleeping, and (x) working. Accuracy results for the activity recognition function of the CASAS SHIB are presented in (46) and vary from high accuracy (cooking with 99%, and personal hygiene activities with 94%) to low accuracy (talking on the phone with 1% and eating with 17%). It is also mentioned that the use of the CASAS SHIB will allow understanding human behavior across many research fields, including health (27).

The Halmstad Intelligent Home (HINT) SHIB approach presented by Lundström et al. (28) focuses on multi-occupancy detection using sensors and an algorithm for binary classification of the occupancy at home of more than one person at the same time. The HINT SHIB is comprised of more than 60 sensors including one SHIB kit. It is mentioned that the HINT SHIB functionality allows: (i) physiological monitoring, safety monitoring, and assistance, (ii) functional monitoring (learning behavior patterns and detecting abnormal behaviors), and (iii) emergency detection and response. The HINT SHIB has capabilities to react and respond to events using actuators. Data were collected from 10 participants who performed eight activities under defined guidelines: (i) go to bed, (ii) use bathroom, (iii) prepare breakfast, (iv) leave house, (v) get cold drink, (vi) office work, (vii) get hot drink, and (viii) prepare dinner. The reported accuracy results obtained using different classification algorithms are high: 82% using a prior knowledge-based algorithm, 75% using a Random Forest algorithm, and 96% using the multi-occupancy detection approach proposed by Lundström et al. (28).

The study presented by Hu et al. (26) evaluates the ability of users to self-install the CASAS SHIB intended for use by elderly people. In this usability study 13 participants, ranging in age from 54 to 86 years old. The average age of the participants was 69.23. Academic qualifications from the participants ranged from high school to Ph.D. The size and number of rooms in the houses of the participants varied. The components of the CASAS SHIB considered were: (i) one installation guide, (ii) one server box, (iii) one relay, (iv) approximately 14 motion sensors, (v) two temperature sensors, and (vi) binary contact sensors in the front door. The evaluation of the usability of the CASAS SHIB installation was carried out by: (i) a post-installation inspection by researchers, and (ii) a questionnaire interview. The criteria considered for how intuitive was the installation of the CASAS SHIB was based on failure rate: (i) a failure rate <10% indicated that the installation process is intuitive, and (ii) a failure rate >35% indicated that the installation process is complicated. It was concluded that some of the CASAS SHIB sensors (motion sensors, area sensors, temperature sensors, and relays) are intuitive to users with no engineering background. Users that had an engineering background considered the installation process of the CASAS SHIB to be intuitive.

The Dem@Home SHIB approach presented by Andreadis et al. (29) is in the form of a framework that integrates a variety of ambient and wearable sensors in addition to interdisciplinary methods of image and semantic analysis. There is a personalization aspect in the Dem@Home SHIB (29), as semantic analysis, activity recognition and problem detection are used to present a profile for every user. The evaluation of the Dem@Home SHIB was carried out for 4 months in four houses where people with mild cognitive impairment or mild dementia were living alone. Sensors were installed in the areas in which users performed most of their daily activities: (i) kitchen, (ii) bathroom, and (iii) bedroom. The activities considered for the activity recognition task were selected with the assistance of a clinical expert: (i) prepare drug box, (ii) cooking, (iii) prepare tea, (iv) watch television, and (v) visit to the bathroom. The evaluation of the activity recognition task was done using precision and recall as evaluation measures. An interesting conclusion from the activity recognition evaluation was that the more atomic and continuous an activity is, the more accurate it is the detection.

The EurValve (Personalized Decision Support for Heart Valve Disease) activity monitoring kit was developed as part of the H2020 EurValve Project, funded by the European Union's Horizon 2020 research and innovation program. It was developed by Pope et al. (30) as a SHIB that could easily be installed in a home by the participant whilst obtaining valuable data around lifestyle and activity levels for medical professionals to review. It consists of one router, four gateways, and one wearable sensor. The kit records physical activity using the wearable and the gateways provide indoor localization data. McConville et al. (47) used the EurValve SHIB rehabilitation monitoring system to collect data from the wrist worn wearable device. The data were collected to evaluate room level indoor localization methods. McConville et al. (47) say how the SHIB approach was low cost and easy to deploy.

From the SHIB approaches presented in this section there are still many aspects that need to be refined, considered, and understood for SHIB approaches to have a more widespread adoption. More studies are needed to learn more about the requirements and needs that will enable elderly users to intuitively install and use a SHIB with little or no help from technical staff.

## Methods

This section presents the SHIB approach developed by the Pervasive Computing Research Center (PCRC) at Ulster University. The design subsection includes a description of the sensors used, as well as how they work in conjunction to collect data from PwD. The implementation subsection presents a case study in which the PCRC SHIB was implemented in a care home.

### Design of PCRC SHIB

**Figure 1:**
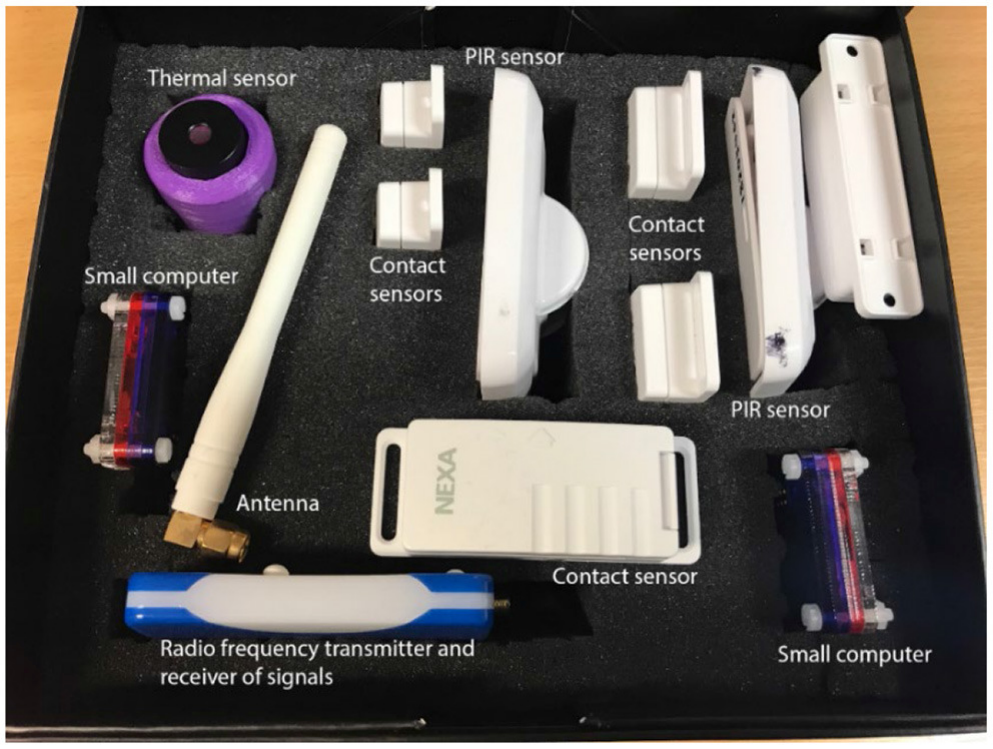
Sensors included in the PCRC SHIB kit.

The PCRC SHIB currently comprises the following types of sensors: (i) thermal vision sensors, (ii) contact sensors, (iii) Passive Infrared (PIR) sensors, and (iv) audio level sensors. The SHIB is presented in a box of 160 x 200 x 80 mm (see Figure 1).

**Figure 2:**
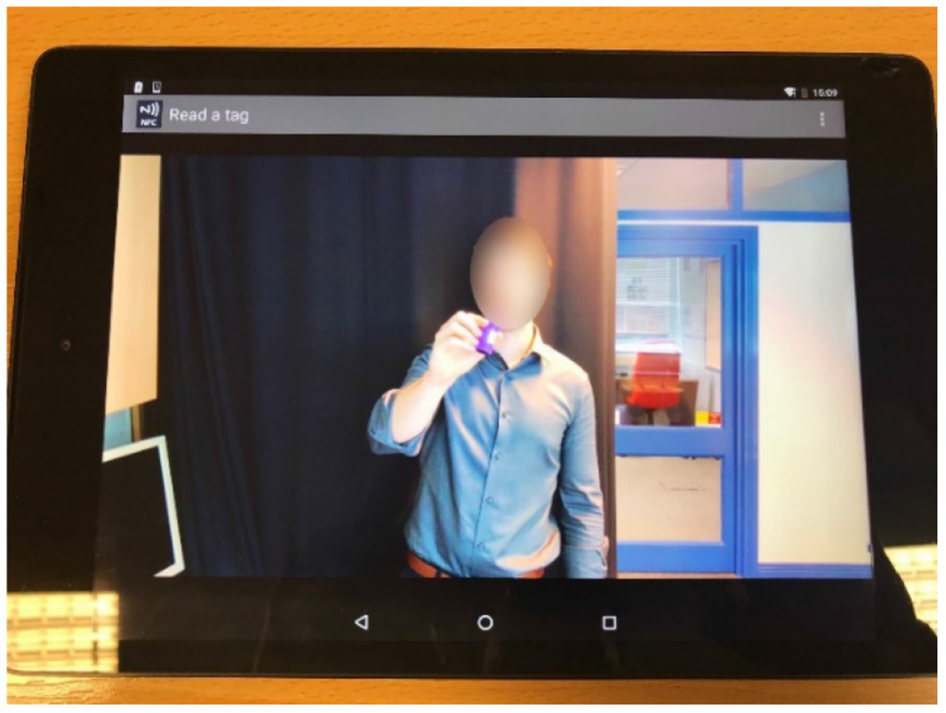
Instructional video activated by NFC tag to demonstrate installation of PCRC SHIB sensors.

In addition to the sensors, the SHIB also contains a small computer to process and send the data from the sensors to the SensorCentral platform (48) (which was developed at PCRC), and an Android tablet with an app to read Near-Field Communication (NFC) tags; these were placed on the sensors to show short videos that describe how to install and utilize the sensors (see Figure 2). The purpose of presenting short instructional videos to the users is to make the SHIB as user-friendly as possible so that users can install and use the sensors by themselves. This SHIB approach also considers offering support to the user in the eventuality that there are issues that the users are not able to solve. Data collected from the PCRC SHIB are stored, processed, and analyzed in SensorCentral. In this sense, data heterogeneity is mitigated by using the timestamps shared by the data points to allow for data fusion.

The thermal sensors used have a resolution of 32 x 31, a 90° by 86° field of view that provides a coverage area of 6 m by 6 m at a height of 2.5 m, and a sample rate of 10 Hz. These thermal sensors are produced by Heimann GMBh and are the HTPA32 model^1^

**Figure 3:**
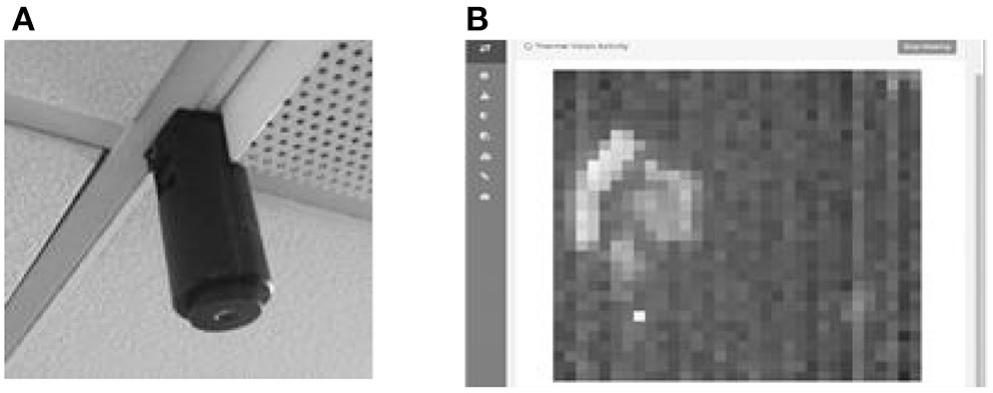
**(A)** Example of prototype thermal sensor attached to the ceiling. **(B)** Example of a matrix generated from data collected from the thermal sensor.

Note that the thermal images captured by the thermal sensor do not have enough resolution to be considered as privacy invasive (49). Figure 3A shows the thermal sensor installed within the PCRC lab and Figure 3B shows a thermal image of a person sitting. The data from the thermal sensor are monitored and collected in SensorCentral. The size, weight, and portability of the thermal sensors allow them to be attached to home surfaces using permanent or temporary mechanisms. In the case of the PCRC SHIB approach, it was deemed that the most adequate place to install the thermal sensors is in a central position in the ceiling of rooms due to: (i) optimization of coverage of the area enabling monitoring the activities of the occupants, (ii) less disruption in terms of distracting or agitating the PwD due to the unobtrusive nature of the installation, and (iii) easy installation (typically, central ceiling positions of rooms provide more access). Deploying the thermal sensor within a central ceiling position allows for an improved Field of View (FoV) due to reducing the number of potential occlusions within the environment. In addition to indicating the presence of a person in a room, the thermal sensor allows the location of the person to be determined in the house or room. The data collected in SensorCentral from the thermal sensors include: (i) a matrix of temperature coordinates per frame, (ii) thermal images generated from the matrix of temperature coordinates for each frame, and (iii) a timestamp.

**Figure 4:**
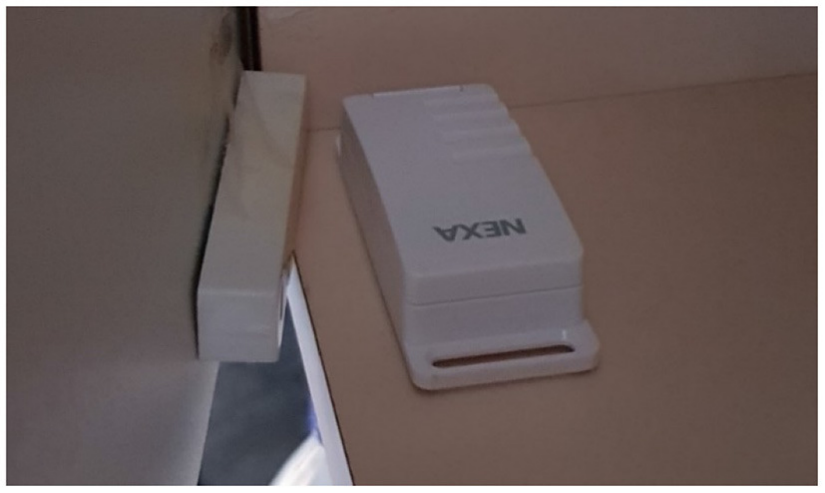
Contact sensors attached to the door and frame of a kitchen cupboard.

Binary contact sensors are a type of sensors that work in pairs by changing states when they are together or separated and can be placed in locations that typically change state such as doors and objects that have a set location to be stored when not in use (e.g., kitchen objects such as a kettle.). The type of contact sensors used were the “NEXA LMST-606 Magnetic Contact Switch”^2^ (see Figure 4), which combines wireless transmitters and magnetic switches. The signals from the contact sensors have two states (“off” or “on”) that change when contact is broken or made between the two components of the sensor. The data from the contact sensors are monitored and collected by the SensorCentral platform (48). The size, weight, self-contained nature, and portability of the contact sensors allow for ease of retrofitting within an environment when compared to that of thermal sensors. Contact sensors can be easily placed on most surfaces and objects around an environment using adhesives. While it is possible to place contact sensors in objects with which the inhabitant of a house has more interaction with, like cups or kettles, for the purposes of the PCRC SHIB the contact sensors are placed on doors of interest in the environment. Thus, the contact sensors give the ability to monitor when person has entered or left the room and allow for inferring and discovering patterns related to the daily life of the PwD. The data collected in SensorCentral from the contact sensors include: (i) event code, (ii) sensor ID, (iii) timestamp, and (iv) name (if defined for that contact sensor, for example, “cupboard1).”

**Figure 5:**
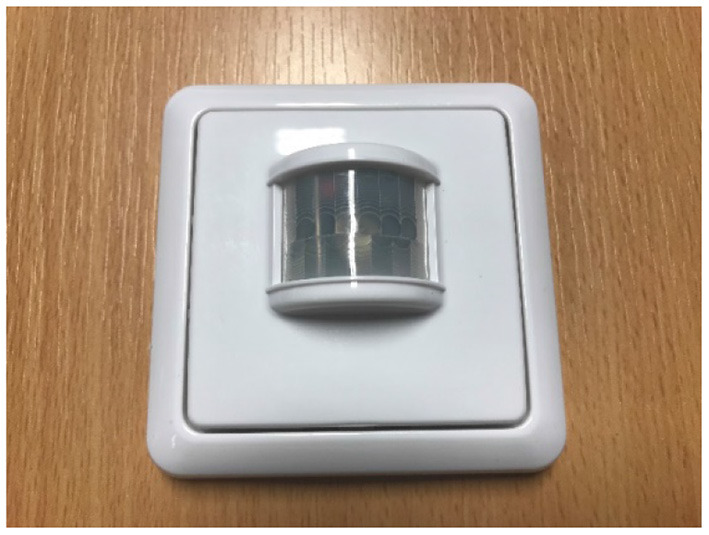
PIR sensor attached to a wood surface.

PIR sensors measure infrared light radiating from moving objects within their FoV. This type of sensors is typically used in alarms for security systems, such as turning lights on automatically when a person is detected within their FoV. The type of PIR sensors used were the “NEXA LMDT-609 Motion Sensor”^3^ (shown in Figure 5), which combine wireless transmitters. The signals from the PIR sensors have two states (“on” or “off”) that change when motion is detected in the proximity of the sensors. The data from the PIR sensors are also monitored and collected by the SensorCentral platform (48). While the PIR sensors are slightly larger than the contact sensors, they still facilitate straightforward and discrete installation within areas of interest within a domicile where a PwD would be monitored. The data collected in SensorCentral from the PIR sensors include: (i) event code, (ii) sensor ID, (iii) timestamp, and (iv) name (if it was defined for that PIR sensor, for example “corridor1”).

**Figure 6:**
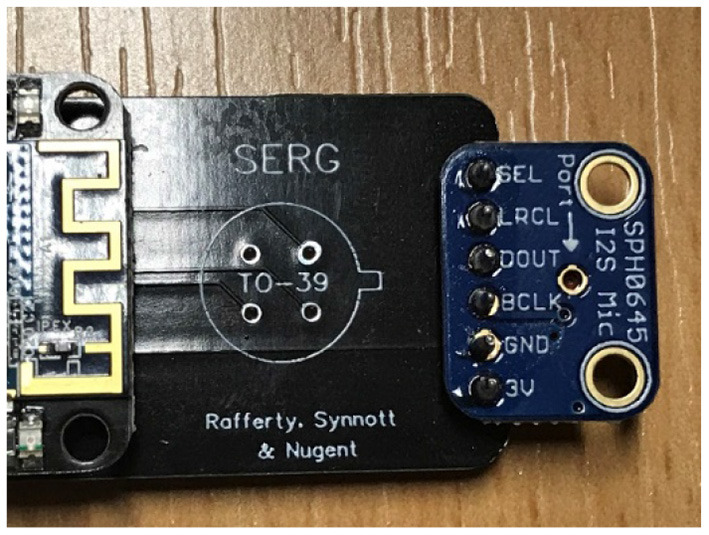
Audio level sensor attached to board.

The audio level sensors used was the “Adafruit 12S MEMS Microphone Breakout”^4^ (shown in Figure 6), which are a type of microphone that detects sound and converts it to a voltage, it has a range of 50 Hz to 15 kHz. Although the microphone is a single mono element, it is sufficient to detect sounds that are used to monitor the activities of the PwD at their homes and rooms. Signals from this sensor are processed by a coupled microcontroller to give a standardized, calibrated, sound pressure level reading in decibels. This type of audio level sensors was used due to their unobtrusive nature and can be used to monitor sound levels of activities of PwD.

**Figure 7:**
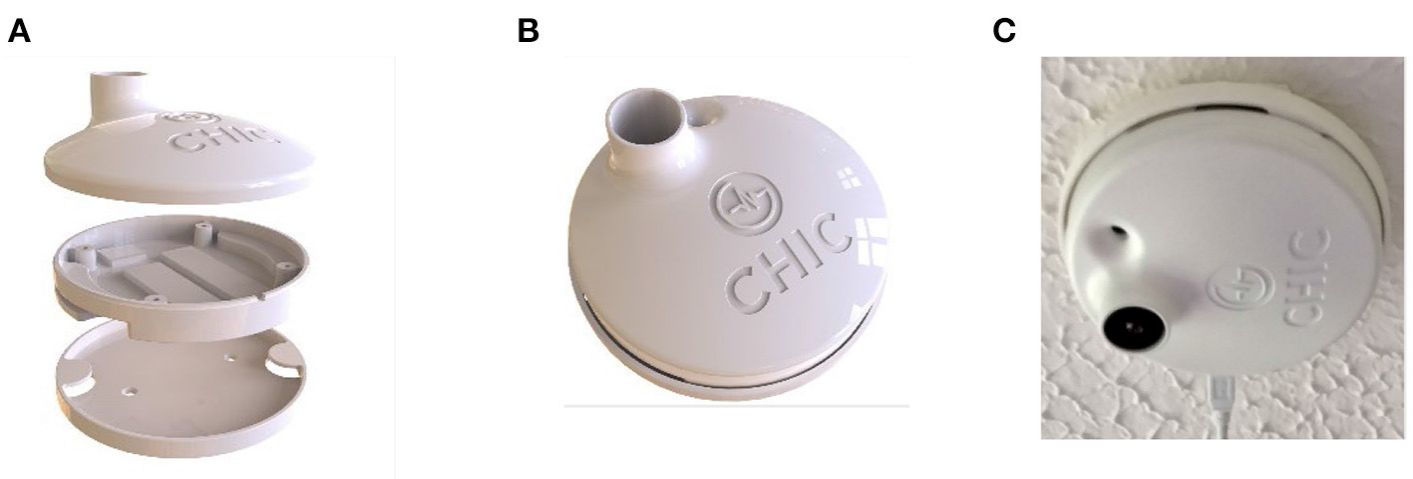
Second version of the integration of the thermal and audio level sensors in a single case: **(A)** components of the case, **(B)** case resembling smoke detector, and **(C)** case installed in the room of a PwD.

Given the size, weight, and portability of the audio level sensors, for the purposes of the PCRC SHIB they were integrated with the thermal sensors within their protective cases that were installed in the ceilings of the rooms where PwD lived. In the first version of the integrated thermal and audio level sensors, the case that contained the sensors was 3D printed in black color. As it will be explained in detail in the next section, it was required to change the color of the sensor housing to white to make them less obvious to PwD. As the initial case designs to hold the thermal and the audio level sensors were not deemed to be the most optimal due to standing out and being distractive for PwD, another design integrating the thermal and audio level sensors in a case that resembled a smoke detector was designed and implemented (Figures 7A–C).

The different versions of the integrated thermal and audio level sensors were attached to the ceiling using a type of industrial adhesive that: (i) was strong enough to keep the case and the sensors in the ceiling while being safe for the PwD, and (ii) did not permanently disfigure the ceiling after removing the case and the sensors. Note that the type of material of the ceiling did not facilitate mechanically affixing the sensors in a semi-permanent way, as it was originally intended. For a permanent deployment of the sensors, it is planned to screw them on the ceiling, where it will still be possible to easily remove them.

The SensorCentral platform facilitates the collection, processing, and analysis of the data collected from diverse types of sensors. The PCRC has already adapted several types of machine learning algorithms to identify patterns that could be inferred as hazards or abnormal behaviors. The data analysis aspect of the PCRC SHIB is beyond the scope of this paper and will be presented comprehensively in a future publication.

### Implementation of PCRC SHIB

The sensors comprised in the PCRC SHIB were tested individually, and as an entire system in the PCRC smart laboratory at Ulster University prior to its deployment in a care home. During the tests performed in the smart laboratory the sensors worked accurately and were shown to be fit for purpose. The next subsections describe the implementation case study of the PCRC SHIB in a care home.

#### Case Study: Belfast Kirk House Care Home

**Figure 8:**
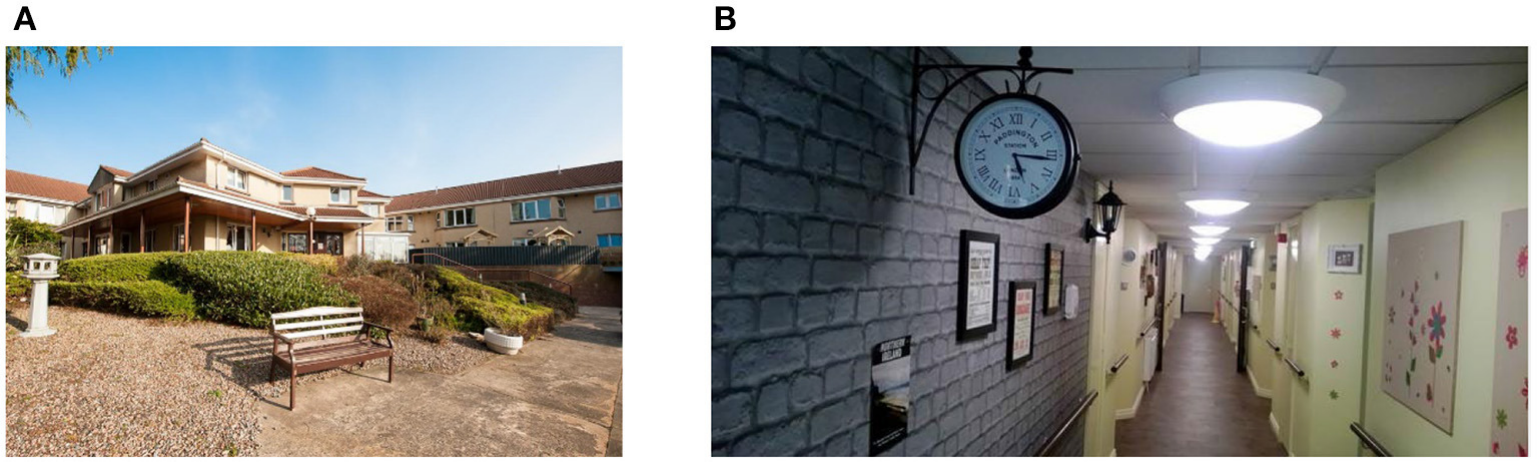
Main entrance of the Kirk House Care Home: **(A)** main entrance, and **(B)** main corridor in the Memory Lane section.

The care home in which the PCRC SHIB approach presented in this paper was installed and tested was Kirk House Care Home in Belfast^5^ (see Figure 8A), this comprises 42 individual flatlets, with nine of them adapted for residents with dementia. The area of Kirk House Care Home in which care is provided for PwD is called “Memory Lane” (see Figure 8B). The trial period lasted 6 months, in which a range of sensors were tested throughout. The sensors included in the PCRC SHIB were installed in the rooms of three PwD to monitor their ADLs. There is a long-standing relationship between the PCRC research group at Ulster University and the management and staff of Kirk House Care Home. This relationship between PCRC and Kirk House Care Home has allowed collaborations like the PCRC SHIB project presented in this paper and the organization of events to engage the public in the use of technology to support elderly people and PwD.

Note that if the SHIB were to become a commercial product, it is intended that the PCRC SHIB will be bought by organizations and individuals that would like the activities of relatives or even their own activities to be monitored to support their health and wellbeing. This could assist with identifying abnormal behaviors that could represent hazards to their well-being or the signs of progressive health problems. For the evaluation of the PCRC SHIB in care homes around the Greater Belfast area, it was required to have in place: (i) ethical approval from the Office for Research Ethics Committees Northern Ireland (ORECNI), and (ii) consent from the PwD and/or their relatives. As it was mentioned in the “Introduction” Section and in relation to the work by Birchley et al. (19) about smart homes and privacy, it is important to consider, where possible and considering the technological limitations, the preferences of the PwD and their relatives in terms of privacy and end-user choices.

Once ethical approval from ORECNI was obtained and staff at Kirk House Care Home had agreed on participating in the PCRC SHIB project, there were many informative talks from the PCRC research team to care home staff, PwD (where possible), and their relatives. The purpose of these informative talks was to describe to the staff, PwD, and their relatives what the PCRC SHIB project entailed and the potential benefits for the PwD living at Kirk House Care Home. During these informative talks, PwD and/or their relatives agreed or denied their consent to take part in the study. PwD and their relatives could opt-out of the study at any time. During these talks it was also clarified that the use of the sensors, the data, the future analysis of the data, and the possible inferences from the collected data were not going to substitute the assistance provided by the caregivers working at Kirk House Care Home. The role of caregivers within the PCRC SHIB study was to ensure that the sensors and the devices used: (i) were not disconnected by PwD, and (ii) were not causing distress to the PwD.

**Figure 9:**
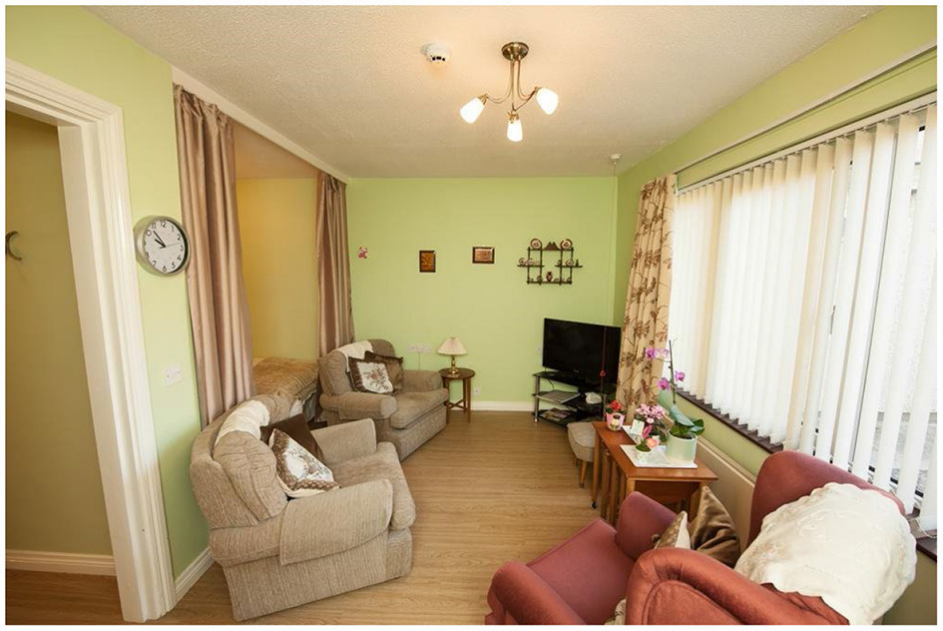
Lounge area of a room in Kirk House Care Home's Memory Lane section.

Once the PwD and their relatives had agreed to take part in the PCRC SHIB study, their rooms within Kirk House Care Home were located in a map showing the architecture of the building to consider the best physical sensor deployment architecture to be installed. The next step was to carry out a physical survey to the building to know more relevant details about it like: (i) thickness of the walls, (ii) location of power plugs and sockets, and (iii) location of telephone sockets. The rooms of the PwD where sensors would be installed were measured and analyzed. The rooms comprised the following areas: (i) bedroom area, (ii) lounge area (see Figure 9), and (iii) bathroom area. Note that the layout of each room varied, so the location of the areas in each room were slightly different. In terms of the expected functioning of the PCRC SHIB system and sensors, there were no evident obstacles or restrictions. These first inspections focused more on the functionality of the PCRC SHIB system and less on the design of the devices and their cases and how they would integrate into the rooms of the PwD.

#### Installation Setup and Tests of PCRC SHIB in Kirk House Care Home

The Wi-Fi enabled router was installed in a central location in the main corridor of the Memory Lane section of Kirk House Care Home (see Figure 8B) where tests indicated to be the optimal location, considering signal strength and communication between the sensors and the router. The router was discreetly placed on top of a bookshelf. To the best knowledge of the PCRC SHIB team, and from what the Kirk House Care Home staff reported, there were no known cases of residents interfering with the router.

After the router was deployed, the sensors in the PCRC SHIB were installed in the rooms of the PwD participating in the study. The installation of the sensors was performed by two suitably qualified researchers from PCRC at times when the PwD were not in their rooms to avoid situations that would cause stress or confusion to the PwD. Staff at Kirk House Care Home supported the PCRC researchers in taking care of the PwD during the installation of the sensors.

Once the sensors were installed in the rooms of the PwD, several communication tests between the installed sensors, the router and SensorCentral were carried out to ensure that the data were being transmitted and stored correctly. During the tests, two researchers from PCRC were at Kirk House Care Home while another PCRC researcher was at Ulster University's Jordanstown campus confirming that the data were being streamed and collected correctly on the SensorCentral platform. The tests highlighted problems with communication between the sensors, the router and the SensorCentral platform, which were related to the strength of the signals sometimes not being received or transmitted correctly. In the cases when there were communication problems, different sensor arrangements were tested to evaluate which was the most reliable. This was determined when there were no communication problems or when they were minimized. When it was deemed that the sensors were installed in the most reliable way, PCRC researchers visited Kirk House Care Home regularly during the trial period (6 months) and one final time after that to present a project debrief to the care staff involved.

## Results

The design, installation, and utilization of the PCRC SHIB in the rooms of PwD at Kirk House Care Home provided the PCRC research team with knowledge and insights related to the challenges and opportunities for future deployments. In general, it was found that the transition from the lab environment to the care home environment presented unexpected challenges that prevented the deployment of a system which was reliable enough to undergo a full-scale deployment. The challenges, opportunities, and lessons learned from the installation and utilization stages of the PCRC SHIB are explained in the following subsections.

### Installation of the PCRC SHIB–Challenges, Opportunities, and Lessons Learned

For this stage of the PCRC SHIB study the expectation was to install sensors in the rooms of service users within dementia units, delirium units, or intermediate care. The sensors to be installed were: (i) thermal sensors, (ii) contact sensors, (iii) PIR sensors, and (iv) audio level sensors. Note that for the sensor's installation during this study the thermal and audio level sensors were integrated within the same protective case. The installation would involve finding the best way to integrate the sensors with the current design and furniture of the room and building, so that it was not distracting or disturbing for service users, particularly those with cognitive impairment because of dementia or delirium.

To facilitate the transition from sensor deployment from the lab environment to real-world trial sites, the sensors were installed in the rooms of a sample of residents with dementia in the Memory Lane section at Kirk House Care Home. It was planned to first establish reliable data collection and processing within this trial site before expanding the deployment to further trial sites. The approach that was followed for installing the sensors prioritized two aspects: (i) safety of PwD, and (ii) adequate installation for data collection and wireless communication with the router. Similar approaches to the ones the PCRC have followed at the PCRC smart environment at Ulster University were followed, considering the different architecture of the care home.

There were two relevant improvements completed with respect to the installation of the sensors: (i) changing the mechanism of attaching the sensors to the ceiling, and (ii) changing the color and shape of the case of the sensors that were attached to the ceiling from black to white. In the first case, after one visit to check the sensors, it was noticed that part of the cables connecting the sensors to the power were starting to detach from the ceiling, therefore a different mechanism using a type of industrial adhesive to attach the sensors and cables to the ceiling was used. The color and shape of the case of the sensors attached to the ceiling was changed from black to white because a PwD was confused about the “black object” in the ceiling. Thus, the case was painted in white color so it would resemble a smoke detector and to match the color of the ceiling.

Sensors to be installed in a care home need to be installed in a way that does not disturb or alter the behavior of PwD. In the case of the sensors that were installed, disguising the case of the thermal and the audio level sensors as a smoke detector helped to prevent PwD from noticing them or to become agitated by them. A detailed survey of the environment in which the sensors will be installed is of key importance. In particular, the surfaces to which the sensors will be installed upon should be inspected to ensure the correct method of fitting.

### Utilization of PCRC SHIB–Challenges, Opportunities and Lessons Learned

In this case the main objective was to have a proper installation of a router that would communicate with the sensors installed in the rooms of PwD. The router would be in the most adequate location to send and receive data to and from the sensors respectively. Additionally, data would be sent to SensorCentral, for further processing and data analysis.

Signal strength tests were completed throughout the Memory Lane section of Kirk House Care Home to identify optimal placement of wireless communication equipment. This communications equipment was installed in a corridor which was identified as the optimal location in terms of signal strength and unobtrusiveness, where the router was in range to the sensors. However, during the time the sensors and the router were installed Kirk House Care Home there were many communication problems. The problems involved: (i) interrupted communication, and (ii) incomplete transmission of sensor data. When the problem was identified, many strategies were tested to try to solve the problem like placing the router in a different location within the Memory Lane section of Kirk House Care Home or using signal repeaters. It seemed that the problem with the communication between the router and the sensors was due to the thickness and the material of the walls, which did not allow for effective communication.

Regarding the location in the care home building to where to place the router for the best communication with the sensors installed, it is necessary to carry out a survey of the building. In the case of Kirk House Care Home, a key factor was the thickness of the walls, which prevented proper and reliable communication between the router and the sensors installed. Similarly, to the considerations regarding the installation of the sensors, the conditions at Kirk House Care Home were quite different from those at the PCRC smart lab at Ulster University. A solution for this would be, to first survey the care home building, and then determine the most appropriate router or supporting devices that allow effective communication. Some of the communication problems encountered could be alleviated with solutions based on: (i) edge computing, (ii) embedded on device machine learning models (reducing data required), and (iii) mesh networking.

## Discussion

This study investigated the challenges and opportunities in the stages of design and implementation of a SHIB approach in a dementia care home environment to monitor the wellbeing of PwD. This study also identified lessons that could support improved future implementations of the approach. The SHIB approach presented in this study was developed at Ulster University's PCRC and was tested as a case study at the Memory Lane section of Kirk House Care Home. Sensors from the SHIB (thermal, contact, PIR, and audio level) were installed in the rooms of three participants with varying degrees of dementia to monitor their ADLs. A router was also installed to communicate with the sensors and to send data to the SensorCentral platform. It was expected to install sensors in the rooms of service users within dementia units, delirium units, or intermediate care.

The findings of this study were considered with respect to the following areas: (i) installation of sensors, and (ii) communication between the sensors. The approach that was followed for installing the sensors prioritized two aspects: (i) safety of PwD, and (ii) adequate installation for data collection and wireless communication with the router.

The challenges with respect to installing the sensors in the care home were related to the transition from sensor deployment in the lab environment to a real-world trial site. An important consideration was that most care home buildings were not originally designed to appropriately install ambient sensors. Despite having surveyed the Kirk House Care Home building at the start of the project, in terms of locating the rooms of the PwD and identifying the most adequate places to install the router and the sensors, there were many challenges during the communication and data collection stages of the project. These challenges included: thickness of walls preventing the adequate communication between the sensors and the router, details in the design of the cases protecting the sensors to avoid confusion in the PwD, and finding the causes and solutions for the periods of time when incomplete data were collected. The solutions implemented to the challenges encountered during the duration of the study were considered as opportunities from where to learn and apply in future iterations and implementations of the PCRC SHIB.

The lessons learned were mainly related to the transition from the sensor deployment from the lab environment to a real-world trial site. In addition to carrying out more comprehensive surveys of the building in which the PCRC SHIB would be installed, more studies about the design of the sensors and their protective cases will be carried out to ensure that PwD are not confused or distressed by the sensors installed in their rooms. There were no questionnaires administered to PwD or to their caregivers. However, some caregivers provided some feedback on the advantages and disadvantages of the PCRC SHIB sensors installed. For example, a carer mentioned that an initial version of the case containing the thermal and audio level sensor was distracting to a PwD because its black color contrasted with the white color of the ceiling, thus, the second version of the case was painted in white. Another consideration will involve using specific types or versions of sensors and equipment to ensure better communication between them, according to the specific architectural features of the care home buildings.

## Conclusions and Future Work

The main findings of this study are: (i) most care home buildings were not originally designed to appropriately install ambient sensors, and (ii) installation of SHIB sensors should be adapted depending on the specific case of the care home where they will be installed. It was acknowledged that in addition to care homes, the homes of PwD were also not designed for an appropriate integration with ambient sensors. Hence, another use of a SHIB approach. This study provided the community with useful lessons, that will continue to be applied to improve future implementations of the SHIB approach. Future work will involve the improvement of the sensors in the PCRC SHIB in terms of an even more specific and suitable configuration as well as the procedure to install them in care homes, regardless of the architecture of the buildings. Note that this will also allow an easier integration and user adoption for the installation and use of the PCRC SHIB approach in the homes of PwD, that are still living independently in their own home or that have the support of their relatives for their care. It is planned to carry out comprehensive tests of the PCRC SHIB system for notifications of hazard and progressive chronic behavior detection to support caregivers. Finally, it is envisioned to present a summary of the PwD ADLs patterns, inferred hazards, and abnormal behaviors in the form of easy-to-interpret dashboards to caregivers and relatives.

## Data Availability Statement

The datasets presented in this article are not readily available because data collected for this study was not collected continuously over a sufficient period of time to be used for data analysis. Data was collected to test the design and implementation of the Smart Home in a Box present in the study. Requests to access the datasets should be directed to Matias Garcia-Constantino, m.garcia-constantino@ulster.ac.uk.

## Ethics Statement

The studies involving human participants were reviewed and approved by Office for Research Ethics Committees Northern Ireland (ORECNI). The patients/participants provided their written informed consent to participate in this study.

## Author Contributions

MG-C, CO, JS, CS, AE, IC, CN, and JR wrote and edited the manuscript. GM contributed to the editing of the manuscript. LL, SM, and AS facilitated access to Kirk House for case study. AE and CO installed equipment at Kirk house. All authors contributed to the article and approved the submitted version.

## Conflict of Interest

GM was employed by The Lava Group. The remaining authors declare that the research was conducted in the absence of any commercial or financial relationships that could be construed as a potential conflict of interest.

## Publisher's Note

All claims expressed in this article are solely those of the authors and do not necessarily represent those of their affiliated organizations, or those of the publisher, the editors and the reviewers. Any product that may be evaluated in this article, or claim that may be made by its manufacturer, is not guaranteed or endorsed by the publisher.

## Footnotes

^1^ https://www.heimannsensor.com

^2^ https://www.clasohlson.com/uk/Nexa-LMST-606-Magnetic-Contact-Switch/Pr364910000

^3^ https://www.clasohlson.com/uk/Nexa-LMDT-609-Indoor-Motion-Sensor/Pr364909000

^4^ https://www.adafruit.com/product/3421

^5^ https://www.kirkhousecarehome.co.uk

## References

[1] World Health Organization. Be Healthy Be Mobile, A Handbook On How to Implement mDementia. Geneva: World Health Organization and International Telecommunication Union (2021). Available online at: https://www.who.int/en/publications/i/item/9789240019966.

[2] Wittenberg R Hu B Barraza-Araiza L Rehill A. Projections of Older People Living With Dementia Costs of Dementia Care in the United Kingdom. CPEC Working Paper 5. London: London School of Economics (2019). Available online at: http://www.yhscn.nhs.uk/media/PDFs/mhdn/Dementia/Bulletin/2019/December%202019/cpec_report_november_2019. pdf

[3] Dawson WD Bangerter LR Splaine M. The politics of caregiving: taking stock of state-level polices to support family caregivers. Public Policy Aging Rep. (2020) 30:62–6. 10.1093/ppar/praa005

[4] Alzheimer's Disease International. Dementia statistics. Available online at: https://www.alzint.org/about/dementia-facts-figures/dementia-statistics (accessed May, 2021).

[5] Kisvetrova H Herzig R Bretsnajdrova M Tomanova J Langova K Skoloudik D. Predictors of quality of life and attitude to ageing in older adults with and without dementia. Aging Ment Health. (2021) 25:535–42. 10.1080/13607863.2019.1705758 31870177

[6] Gotanda H Nuckols T Mori K. Comparison of the quality of chronic disease management between adults with and without dementia. JAMA Netw Open. (2021) 4:e219622. 10.1001/jamanetworkopen.2021.9622 33983400 PMC8120327

[7] Krutter S Schaffler-Schaden D Essl-Maurer R Wurm L Seymer A Kriechmayr C. Comparing perspectives of family caregivers and healthcare professionals regarding caregiver burden in dementia care: results of a mixed methods study in a rural setting. Age Aging. (2020) 49:199–207. 10.1093/ageing/afz165 31875879 PMC7047818

[8] Wojcik D Szczechowiak K Konopka P Owczarek M Kuzia A Rydlewska-Liszkowska I. Informal dementia caregivers: current technology use and acceptance of technology in care. Int J Environ Res Public Health. (2021) 18:167. 10.3390/ijerph18063167 33808644 PMC8003488

[9] Shah HB. Understaffed and Overworked: Poor Working Conditions and Quality of Care in Residential Care Facilities for the Elderly. San Francisco, CA: Publications (2017). p.788

[10] Jayasinghe U Harwin WS Hwang F. Comparing clothing-mounted sensors with wearable sensors for movement analysis and activity classification. Sensors. (2020) 2. 10.3390/s20010082 31877780 PMC6983049

[11] Jaber Al Nahian M Raju MH Tasnim Z Mahmud M Ahad MAR Kaiser S. Contactless fall detection for the elderly. In: Contactless Human Activity Analysis, Intelligent Systems Reference Library 200. Cham: Springer (2021).

[12] Wang J Spicher N Warnecke JM Haghi M Schwartze J Deserno TM. Unobtrusive health monitoring in private spaces: the smart home. Sensors. (2021) 21. 10.3390/s21030864 33525460 PMC7866106

[13] Saleh MM. WSNs and IoT their challenges and applications for healthcare and agriculture: a survey. Iraqi j electr electron eng. (2020) 37–43. 10.37917/ijeee.sceeer.3rd.6

[14] Cosoli G Spinsante S Scalise L. Wrist-worn and chest-strap wearable devices: Systematic review on accuracy and metrological characteristics. Measurement. (2020) 159:107789. 10.1016/j.measurement.2020.107789

[15] Coulby G Clear AK Jones O Godfrey A. Low-cost, multimodal environmental monitoring based on the Internet of Things. Build Environ. (2021) 203:108014. 10.1016/j.buildenv.2021.10801433374270

[16] Voukelatou V Gabrielli L Miliou I Cresci S Sharma R Tesconi M. Measuring objective and subjective well-being: dimensions and data sources. Int J Data Sci Anal. (2021) 11:279–309. 10.1007/s41060-020-00224-2

[17] Casaccia S Revel GM Cosoli G Scalise L. Assessment of domestic well-being: from perception to measurement. IEEE Instrum Meas Mag. (2021) 24:58–67. 10.1109/MIM.2021.951364127295638

[18] Chung J Demiris G Thompson HJ. Ethical considerations regarding the use of smart home technologies for older adults. Annu Rev Nurs Res. (2016) 34:155–81. 10.1891/0739-6686.34.155 26673381

[19] Birchley G Huxtable R Murtagh M Ter Meulen R Flach P Gooberman-Hill R. Smart homes, private homes? An empirical study of technology researchers' perceptions of ethical issues in developing smart-home health technologies. BMC Medical Ethics. (2017) 18:23. 10.1186/s12910-017-0183-z 28376811 PMC5379767

[20] Hall F Maglaras L Aivaliotis T Xagoraris L Kantzavelou I. Smart Homes: Security Challenges and Privacy Concerns. arXiv:2010, 15394. *(arXivorg)*, Computer Science, Cryptography and Security (2020).

[21] Silva JR Macedo ECT Correa YG Medeiros P. A multilayer proposal to a smart home applied to healthcare. Polytechnica. (2021) 4:1–14. 10.1007/s41050-021-00029-7

[22] Fotouhi A Oudi M Gueni B Mabrouk MB. User behavior analysis using IoT data. Nordic and Baltic. JICT. (2021) 2020:263–74. 10.1109/PERCOM.2003.119278327295638

[23] Almeida A Azkune G. Predicting human behaviour with recurrent neural networks. Appl Sci. (2018) 8:305. 10.3390/app8020305

[24] Li Y. Research Direction of Smart Home Real-time Monitoring. In: 2020 International Conference on Computer Engineering and Intelligent Control (ICCEIC). Chongqing: IEEE (2020). p. 220–2. 10.1109/ICCEIC51584.2020.00051

[25] Sanchez VG Pfeiffer CF Skeie N. Methods for Assisting Older People Living Alone. J Sens Actuator Netw. (2017) 6. 10.3390/jsan603001133345612

[26] Hu Y Tilke D Adams T Crandall AS Cook DJ Schmitter-Edgecombe M. Smart home in a box: usability study for a large scale self-installation of smart home technologies. J Reliab Intell Environ. (2016) 2:93–106. 10.1007/s40860-016-0021-y 28936390 PMC5604889

[27] Hayashi V Ruggiero W. Non-invasive challenge response authentication for voice transactions with smart home behavior. Sensors. (2020) 20. 10.3390/s20226563 33212905 PMC7698362

[28] Lundström J De Morais WO Menezes M Gabrielli C Bentes J Sant' Anna A. Halmstad intelligent home-capabilities and opportunities. International Conference on IoT Technologies for Healthcare. (2016) 9–15. 10.1007/978-3-319-51234-1_2

[29] Andreadis S Stavropoulos TG Meditskos G Kompatsiaris I. Dem@Home: ambient intelligence for clinical support of people living with dementia. ESWC. (2016) 357–68. 10.1007/978-3-319-47602-5_49

[30] Pope JR McConville M Kozlowski X Fafoutis R Santos-Rodriguez R Piechocki RJ. SPHERE in a box: practical and scalable EurValve activity monitoring smart home kit. IEEE 42^nd^ Conference on Local Computer Networks Workshops (LCN Workshops) (2017). p. 128–35

[31] Fang Y Lim Y Ooi SE Zhou C Tan Y. Study of human thermal comfort for cyber-physical human centric system in smart homes. Sensors. (2020) 20. 10.3390/s20020372 31936499 PMC7014145

[32] Kidd CD Orr R Abowd GD Atkenson CG Essa IAB MacIntyre E. The aware home: a living laboratory for ubiquitous computing research. International Workshop on Cooperative Buildings. (1999) 191–8. 10.1007/10705432_17

[33] Cook DJ Youngblood M Heierman EO Gopalratnam K Rao S Litvin A. MavHome: an agent-based smart home. IEEE Pervasive Comput. (2002) 1:76–82. Available online at: https://www.utm.my/intrest/2018/11/30/volume-12-issue-2-nov-2018/

[34] Nacer A Marhic B Delahoche L. Smart Home, Smart HEMS, Smart heating: An Overview of the Latest Products and Trends. In: 2017 6^th^ International Conference on Systems and Control (ICSC). Batna: IEEE (2017). p. 90–5.

[35] Mekuria DN Sernani P Falcionelli N Dragoni AF. Probabilistic Logic Reasoning in Multi-agent Based Smart Home Environment. Springer, Ambient Assisted Living, Italian Forum (2019).

[36] Iqbal A Ullah F Anwar H Kwak KS Imran M Jamal W. Interoperable Internet-of-Things platform for smart home system using Web-of-Objects and cloud. Sustain Cities Soc. (2018) 38:636–46. 10.1016/j.scs.2018.01.044

[37] Helal S Mann W El-Zabadani H King J Kaddoura Y Jansen E. The gator tech smart house: a programmable pervasive space. Computer. (2005) 50–60. 10.1109/MC.2005.10727295638

[38] Kamara-Esteban O Pijoan A Alonso-Vicario A Borges CE. On-demand energy monitoring and response architecture in a ubiquitous world. Pers Ubiquitous Comput. (2017) 21:537–51. 10.1007/s00779-017-1014-4

[39] Mano LY. Emotional condition in the Health Smart Homes environment: emotion recognition using ensemble of classifiers. IEEE International Symposium on Innovations in Intelligent Systems and Applications (INISTA). (2018) 1–8. 10.1109/INISTA.2018.846631827295638

[40] Kabir MH Hoque MR Seo H Yang S-H. Machine learning based adaptive context-aware system for smart home environment. Int J Smart Home. (2015) 9:55–62. 10.14257/ijsh.2015.9.11.07

[41] Mokhtar WHW Ismail A. Adoption of smart home technologies features among the homeowners in Hulu Langat, Selangor. INTREST. (2018) 12:9–20.

[42] Brenon A Portet F Vacher M. Arcades: a deep model for adaptive decision making in voice controlled smart-home. Pervasive Mobile Comput. (2018) 49:92–110. 10.1016/j.pmcj.2018.06.011

[43] Hu Y Cook DJ Taylor ME. Study of effectiveness of prior knowledge for smart home kit installation. Sensors. (2020) 20. 10.3390/s20216145 33137911 PMC7663102

[44] Abdulrazak B Helal A. Enabling a Plug-and-play integration of smart environments. In: 2nd International Conference on Information and Communication Technologies. Damascus, Syria (2006). p. 820–5. 10.1109/ICTTA.2006.168447927295638

[45] Katsivelis N Anastasiou A Petropoulou O Lambrou G Giokas K Koutsouris D. Applied technologies and Smart Home applications in the health sector. In: IEEE 30^th^ International Symposium on Computer-Based Medical Systems. Thessaloniki, Greece (2017). p. 544–9. 10.1109/CBMS.2017.48

[46] Cook DJ Crandall AS Thomas BL Krishnan NC. CASAS: a smart home in a box. Computer. (2012) 46:62–9. 10.1109/MC.2012.328 24415794 PMC3886862

[47] McConville R Byrne D Craddock I Piechocki R Pope J Santos-Rodriguez R. A dataset for room level indoor localization using a smart home in a box. Data in Brief. (2019) 22. 10.1016/j.dib.2019.01.040 30740491 PMC6356000

[48] Rafferty J Synnott J Ennis A Nugent CD McChesney I Cleland I. SensorCentral: A research oriented, device agnostic, sensor data platform. In: International Conference on Ubiquitous Computing and Ambient Intelligence. (2017). p. 97–108. 10.1007/978-3-319-67585-5_11

[49] Rafferty J Synnott J Nugent C Morrison G Tamburini E. Fall detection through thermal vision sensing. In: Ubiquitous Computing And Ambient Intelligence. (2016). p. 84–90. 10.1007/978-3-319-48799-1_10

